# Pick the Freshmen Up for a “Healthy Study Start” Evaluation of a Health Promoting Onboarding Program for First Year Students at the Carinthia University of Applied Sciences, Austria

**DOI:** 10.3389/fpubh.2021.652998

**Published:** 2021-04-22

**Authors:** Andrea Limarutti, Marco Johannes Maier, Eva Mir, Doris Gebhard

**Affiliations:** ^1^Department of Health Sciences and Social Work, Carinthia University of Applied Sciences, Spittal an der Drau, Austria; ^2^Department of Human Sciences, University of Education Schwäbisch Gmünd, Shwäbisch Gmünd, Germany; ^3^Independent Researcher, Vienna, Austria; ^4^Faculty of Sports and Health Sciences, Technical University of Munich, Munich, Germany

**Keywords:** students' health promotion, Healthy Universities, employability, outdoor pedagogical intervention, peer to peer approach, sense of coherence, social belonging, self- and social competencies

## Abstract

**Introduction:** Universities are an essential setting for creating health promoting environments. Evidence shows that university life can pose various threats to the students' health. Especially first year students are vulnerable to mental health issues. To support well-being and prevent psychological distress from the first day of studying, onboarding programs are needed to promote the students' health and their self- and social competencies. The study demonstrates a tailored multi-component onboarding intervention program named “Healthy Study Start.” An evaluation of the effectiveness is presented focusing on outcomes regarding the students' sense of coherence (S-SoC), social support, sympathy, the work-related collective and the participative safety (a sub-scale of the team climate) among freshmen at the Carinthia University of Applied Sciences (CUAS, Austria).

**Methods:** For the analyses, a quantitative controlled study design was used and results were measured three times. The intervention group (*n* = 72) was composed of freshmen selected from the bachelor study programs Occupational Therapy, Speech and Language Therapy, Biomedical Science and Radiologic Technology. Freshmen from the bachelor study program Healthcare and Nursing formed the control group (*n* = 81). As the requirements for analyses of variance were not fulfilled, the data had to be analyzed using e.g., Mann-Whitney U-tests.

**Results:** Significant changes (all *p* < 0.016) between the two groups were found between T0/T1, and between T0/T2. Furthermore, changes within the intervention group (all *p* < 0.016) emerged in nearly all outcomes between T0/T1, while within the control group no changes were identified. However, the intervention group had statistically significantly higher values in the majority of outcomes at T1 and T2 compared to the control group.

**Conclusion:** The onboarding program “Healthy Study Start” shows how an initiative at the beginning of their studies can support students in entering a new phase of their lives. The results indicate a positive effect on the students' self- and social competencies. However, students' health promotion is not only an investment for a health conscious university or an enhanced employability. Especially in health-related fields of study, students are future multipliers and play an essential role in implementing health promotion concepts for clients, patients and employees.

## Introduction

In 1995, the World Health Organization (WHO) and the University of Central Lancashire identified universities as an essential setting for creating health promoting environments (1). Since then, the setting has established itself as a health promoting living environment. Exactly 20 years later, in 2015, the latest Charter, the Okanagan Charter (2) called for creating a supportive living environment specifically for the university to promote the health and well-being of students and to support self-competencies. On the one hand, universities are places where students undergo substantial life changes, where opportunities to explore and experiment are offered and the possibility to develop independence and life skills is being given. On the other hand, universities can be seen as a place where students face particular health challenges (3). Indeed, evidence shows an increasing prevalence in mental health issues (4, 5); 12–50% of students meet the criteria for at least one common mental disorder (6). In Austria, 57% of all university students complain to be affected by stress-related challenges, and 48% of the students suffer from mental health issues (7). Grützmacher et al. (8) present nationwide, meaningful and reliable data on students' health situation in Germany. According to their data, 15.6% of the surveyed students show symptoms of a depressive syndrome including loss of joy, interest and energy, and 17.4% suffer from an anxiety disorder. More than a quarter of the surveyed students feel a high level of stress. Exhaustion is experienced by 24.4% of the participants. Furthermore, 22.9% report a sense of loss of importance regarding their studies. Hofmann et al. (9) point out that student-specific burdens include failing exams, coping with university demands, experiencing loneliness, and difficulties in dealing with stress.

Especially the transition from school to university and the associated transition from adolescence to adulthood represent a critical and vulnerable period for young adults. This age span between 18 and 25 years is called “emerging adulthood” (10). Students are in a moral dilemma. On the one hand, starting their university education provides the perfect context for the development of autonomy and the opportunity to establish profound relationships with peers (11, 12). On the other hand, the initial year can be a very stressful experience for students due to new, unprecedented challenges (13). Many factors affect first year students' well-being: change of residence, finding orientation in the new university setting, making new friends, a higher workload or the feeling of competition (9, 11, 12, 14). According to Bruffaerts et al. (6) nearly one third of first year students develop mental health problems during the first 12 months. These problems are associated with lower academic performance. The Austrian Student Social Survey (15) reveals that almost one fifth of first year students suffer from stress-related health issues and 17% from a lack of self-esteem. Besides, 17% report that they find it challenging to organize their studies individually, and 15% complain about depressive moods. Moreover, 14% of the students suffer from existential fears, and 14% are affected by contact difficulties or social isolation.

Evidence points to the fact that most of the psychological distress emerges in the initial year and usually persists throughout the studies' whole duration (16). Students struggle with insufficient time- and self-management, academic demands, lack of confidence, low coping strategies or insufficient capacity to respond to stressful situations (17). To be able to deal with challenging demands, such as the change of residence, orientation within the new university setting, development of new relationships, self-organization, self-efficacy, well-developed social and self-competencies are needed. Strengthened social competencies consolidate the students' ability to form and maintain social relationships and to cooperate with others (18–20). Evidence shows that social support among peers and university friendship groups are the most effective tools for preventing university students' distress (21). Furthermore, social belonging, the feeling of having positive relationships with others, is an individual need (22) which is essential to cope with perceived threats and is related to academic progress, achievement and social acceptance (23). Self-competencies include, for example, self-efficacy, self-management, self-regulation, self-dependence, or stress handling (18–20). To promote those, a strong sense of coherence (SoC) might be useful. From researching a representative sample of the Danish population, Trap et al. (24) have concluded that there is a positive correlation between SoC and self-efficacy. Within the university context, studies show that a higher SoC is related to a better adoption of self-regulated learning strategies, and that students with a higher SoC are more self-regulated in their learning approach (25). Furthermore, better academic performance and social support are associated with a high SoC (26). With a strong SoC, situations are perceived as understandable, meaningful and manageable, and a recovery from stressful experiences is more efficient (27). In order to strengthen the students' sense of coherence (S-SoC), and thus an important resource for health, it is important that university processes can be understood (comprehensibility), that students see their studies as meaningful (meaningfulness), and that they are able to cope with demands (manageability) (28).

Numerous intervention studies have been carried out and published on how to best promote the students' health and well-being and to reduce stressors and maladaptive coping strategies. They focus on specific risk behavior such as alcohol consumption, drug abuse, sexual health issues, smoking, and sleep problems or media consumption (29, 30). There are also resource-oriented interventions to foster resilience in healthcare students, as shown in the review by Kunzler et al. (31), which provide evidence how a resilience training can improve well-being or stabilize mental health. The authors point to the need for further research. Another systematic review issued by Cooley et al. (32) investigated the use of outdoor adventure education in order to facilitate group work in higher education. Studies included in this systematic review mention short icebreakers (e.g., crossing an imaginary minefield, leading students through a “spider's web”) and more challenging activities such as rope courses, rock climbing, caving, trekking and/or orienteering. The outdoor adventure education program was offered to students of different study areas and the majority of the studies were embedded in the degree courses and open for all students. Cooley et al. (32) indicated that these different outdoor education activities might have a positive effect on transferable group skills, because students retained their acquired group work skills when they started higher education. The reviewed studies also indicated a positive effect on team building and some evidence lead to a more positive group environment and more effective group processes. The students' attitudes (e.g., feeling more confident, seeing benefits) toward group work showed a positive development. Lastly, there was evidence that the feeling of social support and integration within the peer group increased.

Resource-oriented approaches for first year students, such as mindfulness training or peer tutoring (16), influence the students' perception concerning their self-care improvement, suggest a reduction of stress related to exams, thus improving scores. de Clercq et al. (33) assessed the effect of two brief social-psychological interventions to promote social-belonging conditions and self-affirmation. The authors describe how the social-belonging intervention has significant effects on outcomes such as social expectations, integration and social studying. However, regarding the intervention concerning self-affirmation only a short-term effect is mentioned.

To sum up, many highly effective intervention approaches exist to promote well-being and health as well as to prevent university students from being exposed to psychological distress. However, a student-tailored health promoting onboarding intervention program, covering social and self-competencies, using different approaches such as outdoor educational, peer to peer and mentoring ones, is still missing. A systematic umbrella review (30) points out a gap in studies concerning health promoting interventions among university students in European countries. The majority of review articles have been published in the US, China and the UK.

Barnett et al. (34) reviewed literature examining the efficacy of psychological interventions (e.g., relaxation, social skills training, attention training, social support, mindfulness, and mediation) regarding the prevention and treatment of mental health disorders in university students. They found out that only 13 out of 84 studies were adapted explicitly for students. Thus, they stress the importance of optimizing interventions for the student population. In terms of a quality development practice of health promotion programs, German speaking countries still lack a sufficient number of published evaluated health promotion interventions to convey substantial information about their benefits and effectiveness (35). Moreover, Cooley et al. (32) indicate that valid questionnaires, strong study designs, analytical procedures and long-term behavior changes are missing.

Summarizing it can be said that in order to enable the students to meet the study-related demands, and to support the students' well-being and prevent them from suffering from possible psychological distress, onboarding programs are needed. It is necessary to promote the students' health and strengthen their self- and social competencies from the first day of studying and consequently over the whole study period and beyond. The present study provides a significant insight into how a student-tailored multi-component onboarding intervention program for first year university students can be composed and contribute to improve the students' health. Additionally, this study procures an important analysis of the effectiveness of the German-speaking regions' onboarding programs.

The development of the onboarding intervention program was based on a mixed methods needs analysis: (1) student-specific burdens and resources, the students' health status, health behavior and interest in health promotion were obtained via a quantitative questionnaire (*n* = 31); (2) resources and strains specific for the study start were investigated within a 4 h “Open Space” session (36). Seventeen students were encouraged within this workshop to develop ideas, based on their personal experiences, about how newly arrived students could be supported by an onboarding intervention program. The results of the quantitative survey and the qualitative “Open Space” session were merged and combined with existing intervention approaches. Based on this procedure, a multi-component onboarding intervention program called “Healthy Study Start” was launched in 2013 to support first year students at the Carinthia University of Applied Sciences [CUAS; (37, 38)] with the aim to promote self- and social competencies of first year students. Since 2015, the program has been an integral part of the curriculum for several health-related study programs. From the moment of implementing the program in 2013, it has continuously been adapted based on the students' feedback. In 2018, a fundamental change of the intervention program was made, due to a relocation. The new location offers attractive indoor and outdoor spaces and the possibility to stay overnight. This environment provides the opportunity to carry out the majority of the program items without a change of location and is ideal for an informal evening program. Particularly the outdoor pedagogical program benefits from the more diverse environment: the original version was mainly based on exercising on high and low ropes courses, whereas the current version offers a wider range of different tasks, spaces and materials used. To examine the effectiveness of the final version of the program, a broad evaluation of the project was launched in 2019.

The objective of the study was to explore the effectiveness of the onboarding intervention program “Healthy Study Start” for first year students of the CUAS concerning the following outcomes:

- students' sense of coherence (S-SoC),
- social support,
- sympathy,
- work-related collective efficacy and
- participative safety (a sub-scale of the team climate).

## Materials and Methods

The onboarding program was evaluated by using a controlled study design. The “Healthy Study Start” project and the accompanying evaluation were carried out in the department of Health Sciences and Social Work at the CUAS between September 23, 2019 and November 30, 2019.

### Study Sample

The intervention program “Healthy Study Start” for freshmen is already an integral part of the curriculum in the bachelor study programs Occupational Therapy, Speech and Language Therapy, Biomedical Science and Radiologic Technology. Thus, the first year students there composed the intervention group (IG, *n* = 72). The onboarding program was implemented for each student class of the four participating study programs. Therefore, the intervention was carried out four times. First-year students attending the bachelor study program Healthcare and Nursing formed the control group (CG; *N* = 81). This study program was best suited as a control group since it also belongs to the health and social studies sector, and an almost comparable number of participants could be collected there. Male and female first year students, from 18 years on, were eligible. Furthermore, the “Healthy Study Start” program and the evaluation are designed to be equally suitable and feasible for students with physical disabilities.

### Contents of the Onboarding Program “Healthy Study Start”

The onboarding program is scheduled for 3 days, includes six components and takes place in the first study week. The program combines different didactical approaches (e.g., peer-mentoring, outdoor pedagogical training) and is based on team teaching. Both lecturers are health scientists with longstanding experiences in practice, research and teaching in the field of health promotion, one additionally with a psychological background and the other with an outdoor pedagogical background.

On the first day, the intervention starts with a 1 h information event presided by the director of the study program in order to give the students a first orientation in their new learning environment (module 1) and aims at promoting the student's comprehensibility. Following this, students participate in a quiz about facts on student's health and are introduced to the background, history and procedure of the ‘Healthy Study Start’ program (module 2). This 90 min long learning session should enable the students to critically deal with students' health related topics, especially focusing on self- and social competencies, which are important skills for their studies, but also for the future employability (39). The aim of module 2 is to enhance the students' awareness for health related topics (e.g., the importance of social support, good self- and time management, coping strategies) and to demonstrate the importance of self- and social competencies for their future working life. The first day ends with 90 min of peer mentoring, developed and conducted by two second-year students of the respective study programs (module 3). Related literature suggests that peer mentoring helps reduce negative effects of stress, provides an access to information about resources at the university, assists with developing skills and promotes social integration (40). It is equally effective in supporting first year students in the transition to university and promotes self- and social competencies (41), which are the aims associated with this third component. The second day takes the students far away from the university campus, to a location in the middle of the woods, where the outdoor pedagogical team training is conducted (module 4). Outdoor based team trainings have been suggested to be feasible and effective in supporting students in developing social competencies and giving students the opportunity to build relationships in an environment away from the daily university routine (42, 43). Some pioneer studies have specifically investigated the implementation of outdoor based team building interventions for first year students. The results indicate that these interventions promote the commitment to the university, facilitate the transition to university life, help newcomers to build positive and trustful relationships with peers and improve their communication and time management skills (44–46). This all-day event starts with a 5 min icebreaker activity and includes five team challenges in the morning, lasting between 30 and 45 min each. In the afternoon, the students have to master a 2 h construction project, go on a hiking tour and finally the program ends with a sound meditation to cool down. The different challenges offer students the opportunity for a collaborative, experiential learning in the following fields: strategizing, planning, decision-making, time management, targeted communication, trust in peers, dealing with frustration and mutual motivation. The acquired group work skills should facilitate a work-related collective efficacy from the beginning of their studies. Module 4 aims to enhance social support, sympathy and participative safety within the group by means of experiencing an intensive cooperation and becoming more familiar with peers. The description of the tasks is outlined in Table 1.

**Table 1:** Task description “Healthy Study Start.”

**Challenge**	**Description**	**Time**
Stepping stones	The team has to overcome a distance of 30 m without touching the ground by using stepping stones. At the same time, they have to fulfill additional tasks such as moving around blindfolded or changing places with other team members. The amount of stepping stones corresponds to the number of participants. For each rule violation (touching the ground) the team loses one stepping stone	30 min
Rope figures	The team members have to spread evenly along a 30-m rope; everybody should hold the rope in their right hand and form a circle together. The game master shows different polygons, (e.g., pentagram) and the team has to copy the figures by moving their bodies but without changing the position of their hand on the rope	30 min
Spider's web	Between two trees, a rope is tensioned zigzag to build a two meters high and two meters wide spider's web with eight openings. All team members stand on the same side of the web and have to pass the spider's web in order to get to the other side. This has to be done by using the openings equally frequently and without touching the web. In case of a rule violation (touching the spider's web), all team members have to go back to the starting point	30 min
Wooden board	The team has to move a wooden stick (three meters long) through five round openings (different diameters) in a wooden board by commonly balancing it on small sticks. It is not allowed to touch the board neither with the long nor with the small sticks. In case of a rule violation, the team has to start again	30 min
Silent sign	The team has to split into two groups, which are positioned at a distance of 25 m. Both groups get one part of a literary quote about studying. The goal is to recombine the two parts of the quote without talking, only by communicating with body movements based on a coding table with the alphabet and its translation into body positions. At the end of the challenge, both groups should have written down the whole literary quote	45 min
Wooden dome	The team has to form two groups, and each receives 150 wooden sticks in three different lengths and connecting pieces with four, five and six openings. The students have to build two wooden, stable domes, three meters high and wide, and they have to use the entire material. During the activity, the two teams are shown different parts of the construction plan for a few seconds and have to exchange the information received to achieve the given goal	120 min
Hiking and talking	During a hiking tour in the woods, the students receive little notes with questions concerning their private life (e.g., hobbies) and about study related topics (e.g., motives for study choice) as conversation starters. They are encouraged to talk in pairs and change the conversation partners several times	120 min

Before the informal evening program starts, module 5 takes place in a relaxed atmosphere: “Meet the Lecturer” provides information about the new living and learning environment, e.g., structure, mission, as well as the people important to know and their roles in the university setting. This component offers some informal tips and tricks from the perspective of an experienced professor. The session aims at making university life more understandable and manageable for students. The second day ends with joint cooking, a campfire and an overnight stay in the location. Finally, the last component, module 6, of the ‘Healthy Study Start’ program happens after breakfast on the third day. During a 90 min long transfer session, students should reflect on the experiences they have made during the outdoor pedagogical training and work on a case study on successful teamwork. Finally, they have to define and sign their ten rules of team working for their 3 years of studies to come. Module 6 aims to promote and strengthen the above mentioned aims of module 4, in order to introduce them into the university setting.

The usual welcome day program of the CUAS, including the ICT (Information and Communication Technology) training, the explaining of the building services and the introduction to the library, is generally offered to freshmen of the intervention and the control group.

For the control group the information period ends here with greetings from the heads of the study area and the study program and a film about nursing. Last time, immediately afterward, one group already started their courses with the first units about “medical terminology” and the other group went home.

### Data Collection

A quantitative test battery was performed at three different times: on the first day of studying (=baseline, T0), at the same time for the intervention and the control group, then directly after the end of the intervention program “Healthy Study Start” (T1) for the intervention group and at the end of the first week for the control group, plus a follow up 2 months later (T2).

Students are informed about the study's aims and data security and that participation is voluntary. Everyone has the right, without giving reasons, to refuse participation. Although the intervention program “Healthy Study Start” is anchored in the intervention group's curriculum and ECTS credits are provided, there is no obligation to participate. ECTS credits can also be achieved in form of substitute performances.

### Outcomes and Measurements

For the analysis of the program's effectiveness the students' sense of coherence (S-SoC), social support, sympathy, work-related collective efficacy and the participatory safety were defined as outcomes. As sociodemographic variables, sex and age were collected.

Students' Sense of Coherence (S-SoC). The 12-item reliable and valid S-SoC scale and its sus-scales comprehensibility (4 items, e.g., “*For me, the Carinthia University of Applied Sciences has clear and transparent structures.”*), meaningfulness (5 items, e.g., “*I have the feeling that the Carinthia University of Applied Sciences is an enrichment for my life.”*) and manageability (3 items, e.g., “*Whenever I am faced with a difficult problem at the Carinthia University of Applied Sciences, I find people who help to solve my problem.”*) were used. According to Brunner et al. (28) Cronbach's alpha is 0.73; for this sample Cronbach's alpha at T0 was 0.80. The S-SoC scale is setting-specific and the items are adapted to the CUAS setting (e.g., “*The CUAS* has clear and transparent structures for me.”). The items could be answered on a seven-point rating scale ranging from 1 = “does not apply at all” to 7 = “applies fully.” Three items had to be reversed before the calculation. For further calculations, the total score, as well as the mean values per sub-scale, could be determined. Higher values indicate a stronger S-SoC.

#### Social Support

The sub-scale of the German questionnaire “Ressourcen und Belastungen von Studierenden” [Resources and demands in Higher Education” (47)] for students was used, ensuring a setting-specific measure. For this sample, Cronbach's alpha at T0 was 0.87. The four items (e.g., “*I easily find someone who informs me or brings me working materials if I cannot come to the university.”*) could be answered on a six-point rating scale: “never,” “rarely,” “sometimes,” “frequently,” “very frequently,” and “always” were offered. High values indicate a high level of perceived social support.

#### Sympathy

The perceived sympathy among the students was measured as an indicator of group sensitivity. Therefore, items of the “Gruppenbefindlichkeitsfragebogen” [Group sensitivity questionnaire (48)] were used. Some items were adapted to the university context [e.g., “I like most of them *(fellow* students) a lot.”]. For this sample, Cronbach's alpha at T0 was 0.85. The items could be answered on a five-point rating scale from 5 = “strongly agree” to 1 = “strongly disagree.” An overall score can be calculated for all items, 4–9 points meaning low sympathy, 10–15 points moderate sympathy, and 16–20 points high sympathy.

#### Work-Related Collective Efficacy

The valid scale (Cronbach's alpha = 0.76, for this sample Cronbach's alpha at T0 = 0.84) is specifically designed for work-related teamwork settings and consists of 8 items (49). All items were adapted to the university context (e.g., “I have confidence that together, *as a student group*, we can manage to meet the project/*university requirements* even under difficult conditions.”). All positively formulated items could be answered on a four-point rating scale (4 = “strongly disagree,” 3 = “rather disagree,” 2 = “partly agree,” and 1 = “strongly agree”). Low values indicate a high level of work-related collective efficacy.

#### Participative Safety

The reliable (Cronbach's alpha = 0.89, for this sample Cronbach's alpha at T0 = 0.87) participative safety scale is a sub-scale of The Team Climate Inventory (50). It combines the following sub-scales: information sharing (Cronbach's alpha = 0.72; 3 items), safety (Cronbach's alpha = 0.65; 2 items), influence (Cronbach's alpha = 0.61; 3 items) and interaction frequency (Cronbach's alpha = 0.79; 4 items). Some items were adapted to the university context [e.g., “Team members *(members of the student group)* feel accepted and understood by the others.”]. The items could be answered on a five-point rating scale from “1 = strongly disagree” to “5 = strongly agree,” respectively “1 = to a very little extent” to “5 = to a very great extent.” For each sub-scale and the overall scale, a score could be calculated on an individual level. High values mean a high level of participative security.

### Statistical Analyses

The employed measures (S-SoC, social support, sympathy, work-related collective efficacy and participative safety) are scales consisting of ordinal items, which are averaged to form scores. At all three measurement time points (at the baseline, immediately after the intervention, and 2 months later), questionnaires were handed out to the participants in both groups, leading to a longitudinal design. Unfortunately, assumptions required for linear methods (e.g., repeated-measures ANOVA) like normality or variables on an interval scale do not hold. Therefore, the data had to be analyzed using a different methodology.

Non-parametric methods (e.g., Mann-Whitney U-tests) can be used to answer cross-sectional questions (e.g., group differences at a specific time point). Due to this study's longitudinal design and scope, the authors opted to calculate differences between time points and to analyze them using nonparametric tests. Differences between time-points (0, 1, 2) are denoted as Δ_01_, Δ_12_, and Δ_02_ for the changes between time-points 0 and 1, 1 and 2, as well as 0 and 2. They are calculated by subtracting the *earlier* from the *later* measurement results, for example, manageability Δ_02_ = manageability_2_–manageability_0_, which yields the following intuitive interpretation: Positive differences signify that manageability_2_ > manageability_0_, therefore manageability-values have increased while negative differences mean that the values have decreased over time.

If either of the two variables contains a missing value, the difference becomes classified as missing. Therefore, differences between medians of variables at specific time-points (cross-sectional; e.g., manageability at t_0_ and t_1_) can differ slightly from the medians of the aforementioned differences (longitudinal; e.g., manageability Δ_01_).

Thus, it can be assessed whether there are changes within a group between time points (testing whether the median differs significantly from 0 for one group) and whether the changes over time are different between groups (test differences between groups).

Since this study aims to assess the effectiveness of the intervention and there are neither prior knowledge nor empirically substantiated assumptions about the effects, all tests are two-sided. Furthermore, although an improvement of students' experiences at the university is at the heart of such interventions, the authors could not rule out unanticipated adverse effects, which would have gone unnoticed in one-tailed tests.

To account for multiple testing, the α = 5% was adjusted using a Bonferroni-correction (adjusted α = 0.016). Effect sizes (η^2^) were computed are considered small for η^2^ < 0.060, medium for 0.060 ≤ η^2^ < 0.140, and large for η^2^ ≥ 0.140 (51).

## Results

### Descriptive Results

A total of 153 freshmen participated in this study, the intervention group including 72 students and the control group 81 students. No dropouts between the occasions were noticed. The majority were women (*n* = 141; missing = 1) and the average age was 22.17 years (*SD* = 5.540).

On the first day of studying (T0), with the exception of social support, no statistically significant differences between both groups were found. After the intervention (T1), the intervention group found university life significantly more manageable than the control group. No significant differences were identified between the two groups immediately after the intervention regarding the sub-scales meaningfulness, comprehensibility and social support. However, 2 months later (T2) the intervention group had a statistically significant (all *p* < 0.005) higher feeling of comprehensibility and social support than the control group. After the intervention, the intervention group felt a significantly higher sympathy toward each other than the control group.

Furthermore, the intervention group had a statistically significantly higher sense of safety and influence than the control group. Additionally, the interaction frequency and the feeling of participative safety were significantly higher within the intervention group after the intervention. Two months later (T2) the intervention group had a statistically significantly (all *p* < 0.005) better feeling of manageability, sympathy, work-related collective efficacy, participatory safety, and information sharing, safety, influence and interaction frequency (Table 2).

**Table 2:** Participants' medians on the outcomes and group differences at all three measure points.

	**Baseline (T0)**			**After Intervention (T1)**			**Two months later (T2)**		
	**IG**	**CG**		**IG**	**CG**		**IG**	**CG**	
	**Median (IQR)**	**Median (IQR)**	***p***	**Median (IQR)**	**Median (IQR)**	***p***	**Median (IQR)**	**Median (IQR)**	***p***
**S-SoC**									
Manageability	5.33 (4.33–5.67)	5.00 (4.67–5.67)	0.473	5.33 (4.92–6.00)	5.00 (4.33–5.67)	**0.021**	5.00 (4.33–5.92)	4.33 (3.67–5.33)	**0.001**
Meaningfulness	5.20 (4.60–5.60)	5.40 (4.60–5.80)	0.126	5.20 (4.60–5.60)	5.20 (4.25–5.80)	0.804	5.00 (4.45–5.80)	4.80 (4.00–5.40)	0.057
Comprehensibility	5.00 (4.50–5.75)	5.00 (4.50–5.50)	0.457	4.50 (4.00–5.525)	4.75 (4.25–5.50)	0.216	4.75 (3.75–5.25)	4.00 (3.00–4.75)	**<0.001**
Social support	3.50 (2.50–4.44)	3.75 (3.00–4.81)	**0.018**	4.00 (3.25–4.50)	3.75 (3.25–4.63)	0.480	4.75 (4.25–5.25)	4.25 (3.50–5.00)	**0.002**
Sympathy	14.00 (12.00–16.00)	13.00 (12.00–16.00)	0.566	17.50 (16.00–19.00)	14.00 (12.50–16.00)	**<0.001**	18.00 (16.00–19.75)	16.00 (13.00–17.00)	**<0.001**
Work–related collective efficacy^*^	1.83 (1.50–2.00)	2.00 (1.50–2.17)	0.236	1.50 (1.17–1.83)	2.00 (1.50–2.00)	**<0.001**	1.67 (1.167–2.00)	2.00 (1.67–2.17)	**0.003**
**Participative safety**									
Information sharing	11.00 (9.00–12.00)	11.00 (10.00–12.00)	0.368	12.00 (11.00–14.00)	11.00 (9.00–13.00)	**<0.001**	13.00 (12.00–14.00)	12.00 (10.00–14.00)	**<0.001**
Safety	8.00 (7.00–9.00)	8.00 (7.00–9.00)	0.967	8.50 (8.00–10.00)	8.00 (6.00–9.00)	**<0.001**	9.00 (8.00–10.00)	8.00 (7.00–9.00)	0.050
Influence	11.00 (9.00–12–00)	11.00 (9.00–13.00)	0.268	12.00 (11.00–13.00)	11.00 (9.00–13.00)	**0.005**	14.00 (12.00–15.00)	12.00 (10.00–13.00)	**0.044**
Interaction frequency	13.00 (11.00–16.00)	14.00 (12.00–16.00)	0.128	16.00 (14.00–18.00)	13.00 (12.00–16.00)	**<0.001**	17.00 (15.00–18.00)	15.00 (11.25–16.00)	**<0.001**
Overall P.s.^**^	43.00 (37.00–48.00)	45.00 (38.50–50.00)	0.189	48.00 (45.00–53.00)	42.50 (36.00–49.75)	**<0.001**	53.50 (48.00–56.25)	47.00 (41.00–52.00)	**<0.001**

*Numbers in bold indicate p < 0.05*.

*Data were expressed as median (IQR) at the baseline (T0), after the intervention (T1) and 2 months later (T2) for the intervention group (IG) and the control group (CG)*.

*Mann-Whitney U-test: p-Values for group differences*.

* *Low values indicate a high level of work-related collective efficacy*.

** *For the Participative safety scale an overall score was calculated (Overall P.s.)*.

### Changes in S-SoC, Social Support, Sympathy, Work-Related Efficacy and Participative Safety Within the Intervention Group and the Control Group

A statistically significant change within the intervention group was found in all outcomes, excepted meaningfulness, between the baseline (T0) and after the intervention (T1). Within the control group, no statistically significant changes emerged (Δ_01_). Overall, the values for manageability and meaningfulness remained constant for the intervention group while they significantly decreased within the control group from T0 to T2 (Δ_02_). While the intervention group started with a significant decline in the S-SoC sub-scale manageability from the baseline to after the intervention, there was no statistically significant change between T1 and T2 (Δ_12_). The control group, however, did not show a significant change after the baseline (T0), but had a substantial drop between T1 and T2 and throughout the study (Δ_02_). While social support remained constant for the control group, it statistically significantly increased within the intervention group after the intervention (T1). Furthermore, there was a convincing increase within the intervention group over the duration of the study. The sympathy toward each other increased statistically significantly more within the intervention group than in the control group over the duration of the study (Δ_02_). The level of the work-related collective efficacy remained constant for the control group from T0 to T1 while it substantially increased within the intervention group from T0 to T1 and across all measurement points (Δ_02_). While the values within the sub-scale information sharing remained constant for the control group, the values for the intervention group increased statistically significantly between T0 and T1. Over time, from T0 to T2, the values within the sub-scale information sharing increased substantially within the intervention group and the control group. No statistically relevant changes were found for the control group for the sub-scales safety, influence, interaction frequency, and overall scale participative safety across all measurement points (Δ_02_). Whereas, for the intervention group significantly increases within the sub-scales safety, influence, interaction frequency and the overall scale participative safety from T0 to T2 were identified (Table 3).

**Table 3:** Changes in S-SoC, social support, sympathy, work-related collective efficacy, and participative safety within the intervention group and the control group.

	**Median difference between** **T0 and T1 (Δ_01_)**				**Median difference between** **T1 and T2 (Δ_12_)**				**Median difference between** **T0 and T2 (Δ_02_)**			
	**IG**		**CG**		**IG**		**CG**		**IG**		**CG**	
	**Δ_01_**	***p***	**Δ_01_**	***p***	**Δ_12_**	***p***	**Δ_12_**	***p***	**Δ_02_**	***p***	**Δ_02_**	***p***
**S-SoC**												
Manageability	0.33	**0.012**	−0.33	0.087	−0.33	0.020	−0.33	**0.002**	0.00	0.862	−0,67	**<0.001**
Meaningfulness	0.20	0.165	−0.20	0.127	0.00	0.559	−0.20	**0.003**	0.10	0.982	−0.40	**<0.001**
Comprehensibility	−0.50	**<0.001**	0.25	0.501	0.00	0.311	−1.00	**<0.001**	−0.50	**0.001**	−1.25	**<0.001**
Social support	0.50	**<0.001**	0.00	0.946	0.75	<0.001	0.50	**<0.001**	1.25	**<0.001**	0.25	**0.008**
Sympathy	4.00	**<0.001**	0.00	0.067	0.00	0.581	1.00	0.083	3.00	**<0.001**	1.00	**0.005**
Work-related collective efficacy^*^	−0.33	**<0.001**	0.00	0.946	0.00	**0.016**	0.00	0.196	0.00	**0.009**	0.00	0.448
**Participative safety**												
Information sharing	1.50	**<0.001**	0.00	0.845	1.00	0.101	1.00	**0.001**	2.00	**<0.001**	1.00	**0.002**
Safety	1.00	**<0.001**	0.00	0.305	0.00	0.971	1.00	0.051	0.50	**0.001**	0.00	0.287
Influence	1.00	**<0.001**	0.00	0.728	1,00	**<0.001**	0.00	0.115	3.00	**<0.001**	0.00	0.434
Interaction frequency	3.00	**<0.001**	0.00	0.093	1.00	0.058	1.00	0.077	3.00	**<0.001**	0.00	0.790
Overall P.s.^**^	6.50	**<0.001**	0.00	0.380	2.00	**0.002**	2.00	0.021	9.00	**<0.001**	2.00	0.047

*Numbers in bold indicate adjusted p < 0.016*.

*The table presents median differences (Δ) between baseline (T0) and after the intervention (T1), after the intervention (T1) and the follow-up (T2) and baseline (T0) and follow-up (T2) for the intervention group (IG) and control group (CG)*.

*Mann-Whitney U-test: p-values for differences within the IG and control group CG from T0 to T1, T1 to T2, and from T0 to T2*.

* *Neg. values means an increase of work-related collective efficacy*.

** *For the Participative safety scale an overall score was calculated (Overall P.s.)*.

### Changes Between Intervention and Control Group in S-SoC, Social Support, Sympathy, Work-Related Efficacy and Participative Safety Over Time

Table 4 shows significant changes and effect sizes between the intervention and the control group between the measurement points. The Mann-Whitney U-tests elaborated substantial changes (all *p* < 0.016) between the groups from T0 to T1 in sympathy, the participatory safety and the sub-scale interaction frequency with strong effect sizes and medium effect sizes for social support, work-related collective efficacy, the sub-scales information sharing, safety and influence. Small effect sizes were found for manageability and comprehensibility, whereas no significant changes emerged within the sub-scale meaningfulness. Across all measurement points, from T0 to T2, statistical changes between the intervention and the control group with strong effect size were registered in the sub-scale influence and medium effect sizes in manageability, comprehensibility, social support, sympathy, the overall scale participatory safety and the sub-scales safety, influence and interaction frequency. Regarding meaningfulness, only a small effect size between the changes was detected.

**Table 4:** Changes in S-SoC, social support, sympathy, work-related collective efficacy, and participative safety between the intervention and control group.

	**Baseline (T0)**		**After Intervention (T1)**		**Two months later (T2)**		**Changes_TOT1** **between IG** **and CG**		**Changes_T1T2** **between IG** **and CG**		**Changes_T0T2** **between IG** **and CG**	
	**IG**	**CG**	**IG**	**CG**	**IG**	**CG**						
	**Median** **(IQR)**	**Median** **(IQR)**	**Median** **(IQR)**	**Median** **(IQR)**	**Median** **(IQR)**	**Median** **(IQR)**	***p***	**ES**	***p***	**ES**	***p***	**ES**
**S-SoC**												
Manageability	5.33 (4.33–5.67)	5.00 (4.67–5.67)	5.33 (4.92–6.00)	5.00 (4.33–5.67)	5.00 (4.33–5.92)	4.33 (3.67–5.33)	**0.003**	0.059	0.348	0.006	**<0.001**	0.087
Meaningfulness	5.20 (4.60–5.60)	5.40 (4.60–5.80)	5.20 (4.60–5.60)	5.20 (4.25–5.80)	5.00 (4.45–5.80)	4.80 (4.00–5.40)	0.033	0.031	0.086	0.020	**0.005**	0.050
Comprehensibility	5.00 (4.50–5.75)	5.00 (4.50–5.50)	4.50 (4.00–5.525)	4.75 (4.25–5.50)	4.75 (3.75–5.25)	4.00 (3.00–4.75)	**0.013**	0.041	**<0.001**	0.129	**0.001**	0.069
Social support	3.50 (2.50–4.44)	3.75 (3.00–4.81)	4.00 (3.25–4.50)	3.75 (3.25–4.63)	4.75 (4.25–5.25)	4.25 (3.50–5.00)	**0.001**	0.077	0.081	0.020	**<0.001**	0.123
Sympathy	14.00 (12.00–16.00)	13.00 (12.00–16.00)	17.50 (16.00–19.00)	14.00 (12.50–16.00)	18.00 (16.00–19.75)	16.00 (13.00–17.00)	**<0.001**	0.171	0.237	0.009	**<0.001**	0.098
Work-related collective efficacy^*^	1.83 (1.50–2.00)	2.00 (1.50–2.17)	1.50 (1.17–1.83)	2.00 (1.50–2.00)	1.67 (1.167–2.00)	2.00 (1.67–2.17)	**0.002**	0.064	0.664	0.001	0.30	0.032
**Participative safety**												
Information sharing	11.00 (9.00–12.00)	11.00 (10.00–12.00)	12.00 (11.00–14.00)	11.00 (9.00–13.00)	13.00 (12.00–14.00)	12.00 (10.00–14.00)	**<0.001**	0.132	0.163	0.013	0.028	0.032
Safety	8.00 (7.00–9.00)	8.00 (7.00–9.00)	8.50 (8.00–10.00)	8.00 (6.00–9.00)	9.00 (8.00–10.00)	8.00 (7.00–9.00)	**<0.001**	0.073	0.028	0.031	0.130	0.015
Influence	11.00 (9.00–12.00)	11.00 (9.00–13.00)	12.00 (11.00–13.00)	11.00 (9.00–13.00)	14.00 (12.00–15.00)	12.00 (10.00–13.00)	**0.001**	0.077	0.040	0.027	**<0.001**	0.157
Interaction frequency	13.00 (11.00–16.00)	14.00 (12.00–16.00)	16.00 (14.00–18.00)	13.00 (12.00–16.00)	17.00 (15.00–18.00)	15.00 (11.25–16.00)	**<0.001**	0.222	0.639	0.001	**<0.001**	0.131
Overall P.s.^**^	43.00 (37.00–48.00)	45.00 (38.50–50.00)	48.00 (45.00–53.00)	42.50 (36.00–49.75)	53.50 (48.00–56.25)	47.00 (41.00–52.00)	**<0.001**	0.217	0.947	0	**<0.001**	0.131

*Numbers in bold indicate adjusted p < 0.016*.

*Data were expressed as median (IQR) for baseline (T0), after the intervention (T1) and the follow-up (T2)*.

*Mann-Whitney U-test: p-value for changes between the intervention group (IG) and the control group (CG) from T0 to T1, T1 to T2, and from T0 to T2*.

*ES = effect size (η^2^): small = 0.010 ≤ to < 0.060, medium = 0.060 ≤ to < 0.140, and large = > 0.140*.

* *Low values indicate a high level of work-related collective efficacy*.

** *For the Participative safety scale an overall score was calculated (Overall P.s.)*.

## Discussion

The onboarding program “Healthy Study Start” shows how an initiative at the beginning of their studies can help students manage this new phase of their lives. It features a clear and conceptual framework and follows a “key principle” of health promotion defined by the WHO (52): the target group's participation. The “Healthy Study Start” intervention program engages students to participate in its development actively. So modules, tailored to the needs of the target group, were conceptualized and students' resources are used and the intervention program's acceptance can be increased by involving the individuals concerned (53). The different didactical approaches (e.g., peer to peer approach, outdoor pedagogical approach) enable students to engage in self-directed, active and interactive learning processes to support self- and social competencies, which promote their self- and professional development (54). Another strength of this onboarding program is the team-teaching approach, with two health promotion experts, one in outdoor educational training and the other in psychology supervising the “Healthy Study Start.” To the best of the authors' knowledge, this is the first controlled study to evaluate a health promoting onboarding program for first year students concerning self- and social competencies, especially S-SoC, social support, sympathy, work-related collective efficacy and the participative safety. Statistically significant differences between the intervention and the control group and statistically significant changes within the intervention group indicate positive effects on the students' self- and social competencies. Statistically significant changes (all *p* < 0.016) between the intervention group and the control group from T0 to T1 were found in manageability, comprehensibility, social support, sympathy, work-related collective efficacy, the participatory safety scale and all sub-scales with medium to large effect sizes. Over time, from T0 to T2 statistically changes between the intervention and the control group were identified in the sub-scale influence with strong effect size and medium effect sizes in manageability, comprehensibility, social support, sympathy, the overall scale participatory safety and the sub-scales safety, influence and interaction frequency. Compared to the control group the intervention group showed significant positive changes within all outcomes, with the exception of meaningfulness (no change was found) and comprehensibility, whose numbers decreased between T0 to T1. An explanation for no statistical changes regarding meaningfulness can be found in the students' study motives. It can be assumed that students choose their line of studies based on their huge desire to engage in a “meaningful profession” and so, due to the limited number of university places, they have to deal with this issue intensively during the admission procedure (55, 56). Another reason could be that the “Healthy Study Start” program promotes the overall S-SoC, whereas there is no tailored intervention to promote meaningfulness. Over time, within the intervention group, positive changes in social support, sympathy, the overall scale participatory safety and the sub-scales occurred. However, in a cross-sectional comparison at the follow up, the intervention group showed statistically significant higher values in nearly all outcomes. These results suggest that the intervention program had a positive effect on the S-SoC scales manageability and comprehensibility. This might especially be connected to the 1 h information event with the director of the study program, the “Meet the Lecturer” event and the reflection and transfer session, because these offers combined may lead to a more detailed discussion about university structures and processes and an intensive confrontation with comprehensibility and manageability within the university setting. It is widely accepted that a strong SoC is a psychological resource that strengthens the individual's competence to deal with environmental strains and stressful situations (57). Evidence shows positive associations between a strong SoC and academic success and achievement (25, 58, 59), social support (25) and adaptive coping behavior (60). Hence, universities should invest in identifying students with low SoC, using a setting-specific measurement such as the S-SoC scale (28) to offer early and timely health promoting interventions. Dooris et al. (61) point out that investigating health and well-being in the university setting should not be done without addressing health needs and problems with a salutogenic focus. Furthermore, the predominantly positive results suggest that the program is a meaningful and interesting method for students to develop social competencies and to promote a trusting climate within the student group. The onboarding program “Healthy Study Start” follows a multifaceted didactical approach including an outdoor pedagogical, a peer to peer and a team-teaching approach. Outdoor pedagogical trainings offer a good opportunity for students to test their ability to cooperate in a setting far away from the university. Furthermore, they enlarge already existing competencies, helping to reflect on weaknesses and to experience the satisfaction of doing things in a group (42). Lastly, they demonstrate that the use of a peer to peer approach provides social connections with other students, which in turn has a positive effect on the sense of belonging, the development of social skills, enhancing the identification with the university context, getting information about resources on campus and academic success (40). Peer to peer support (e.g., peer coaches or peer mentoring) can help to strengthen self-efficacy, support study strategies, improve study habits over time or to overcome study related demands (62). The statistical decrease of the control group's manageability values could be explained by the lack of personal competencies and social support by fellow students to cope with high study-related demands. Bengel et al. (63) report that manageability includes the feeling of having own resources and competencies and the belief that other people can help overcome difficulties. Therefore, manageability improves with social support, which might be essential to cope effectively with stressors. Furthermore, the supervised onboarding program with a team-teaching approach can also positively contribute to the intervention group results. The review of Conely et al. (64) identified that supervised skills training programs were far more effective than others with regard to outcomes including stress, general psychological distress, social or emotional skills, self-perception or academic adjustment.

For the mentioned approaches, positive associations with self- and social competencies have been found in different studies. For instance, Wolfe and Kay (46) state in their study that a first year student's participation in an outdoor orientation program results in a higher commitment to the university, a more successful transition to university life, an emotional, social, and personal growth and positive relationships with others. Bell et al. (65) reviewed 25 published studies examining outdoor orientation programs and conclude that such programs support a sense of belonging among students and healthy peer connections. Furthermore, Herrmann-Werner et al. (66) find that a Tandem Program reduces perceived stress and improves the ability to work in a team within medical students. The systematic review of Akinla et al. (67) analyzed near-peer mentoring programs for first year medical students and identify near-peer mentoring as a promising intervention concerning professional and personal development, stress reduction and ease of transition. Within the onboarding program “Healthy Study Start” all successful approaches are used in combination, although it is difficult to conclude which approach is effective and influences which outcome.

### Contribution to the Field of Students' Health Promotion

This study suggests that the participation of the target group and the combination of a multifaceted didactical approach targeting self- and social competencies may be a promising strategy to promote health and well-being among university students. Besides, this health promoting onboarding program may lead to more understanding of how health and well-being can be promoted within the setting. The support of a healthy personal and social development, the guarantee of a healthy and sustainable working environment, the encouragement of a wider academic interest and the permanent engagement in health promotion are objectives of a Health Promoting University (68). Through the curricular anchoring of the onboarding program “Healthy Study Start” into some degree programs and the associated commitment to invest in student health, first steps toward the Healthy University approach have been set. In addition, the focus on developing self- and social competencies and the evaluation of the program contribute to Healthy Universities. However, it is not solely in the interest of a healthy university that the promotion of personal and social skills among students is implemented. The focusing on the students' self- and social competencies is also in line with the requirements of employability by the European Higher Education Area (Bologna Process) (69). While the term “employability” was minimized to job-relevant opportunities for graduates some time ago, nowadays this term encompasses the ability to acclimatize in a dynamic and transforming labor market and beyond (70). The Yerevan Communiqué (71) advocates that necessary competencies should be trained during the period of studies in order to qualify for a long-term and successful position in the labor market and beyond. The “Healthy Study Start” program follows these requirements and enables students at the beginning of their studies to reflect on their resources, the study-related demands, and to deepen associated key competencies. Focusing on self- and social competencies (e.g., cooperation and communication, teamwork, self-regulated learning and self-awareness) plays a major role in this context (19). Universities are required to promote them in order to support the students' health and ability to study (72). The need for well-developed self- and social competencies has especially become apparent under the special COVID-19 conditions. Students suffer due to social distancing and lack of social support (73, 74). Changing learning environments, particularly a focus on E-Learning, can lead to difficulties with an effective study organization (73). Especially students from healthcare-related study programs are suffering (75). Therefore, it seems even more important to provide adequate support for these students (76). In order to offer students a healthy study start despite the COVID-19 restrictions, the onboarding program has been adapted and adjusted to the current conditions (e.g., no overnight stay, using a corona hygiene sanitation protocol, using materials that are suitable for disinfection). However, the program was adapted to a unique situation and had no impact on this evaluation; as soon as circumstances change, the evaluated “Healthy Study Start” program will be provided again as per the description. Due to the importance of interventions supporting self- and social competencies and health promotion in general, they should not be limited to the beginning of a study program but rather need to be offered throughout the whole study duration. This commitment can guarantee sustainability in working for the students' health and an improvement for the study situation. For this reason, in March 2019, another project named KukiS-Toolbox (a German project called “Kompetent und kohärent im Studium-Toolbox” was designed to enhance self- and social competencies among students) started at the CUAS. It focuses on strengthening the dimensions of S-SoC, on promoting group support and the sense of belonging by developing learning and teaching materials, targeting full-time and part-time students. The materials are available for students and staff. Further, a “Student Health Advisory Board” will be set up, so students can voice health issues concerning their communities and participate in the design of health promoting processes within the university, in this case within the CUAS. As a supportive living environment promoting a student health management (77), the university has still not sufficiently arrived in the focus of attention in Austria, compared to other German-speaking and international areas. However, intervention programs like the ‘Healthy Study Start’, the project “KukiS-Toolbox” and the intended “Student Health Advisory Board” send a first signal for an effective students' health management (78) in Austria. Furthermore, it should be noted that health promotion for students is not exclusively an investment in students' health and well-being during their study period. If they develop awareness for health promotion topics during their studies, they can take on a pioneering role in their future working lives; they are considered multipliers for health promotion (79). Especially in health-related fields of study, students, as future multipliers in the healthcare sector, can play a significant role in workplace health promotion for clients, patients and employees (80).

### Limitations

We acknowledge some limitations to the study. Due to the sample population, some discovered effects might be attributable to the specific setting (CUAS students). More data would permit a more comprehensive analysis. Bias could occur because the control group consists of nursing students only, compared to the intervention group, which comprises a diverse set of students. Furthermore, a gender bias is possible, because a large proportion of female students from health-related study fields participated in the study. The students' perceptions, which could be influenced by numerous factors at the time of completing the questionnaire, may under- or overestimate the actual knowledge and skills acquired.

Moreover, there could be different co-variables, e.g., the fact that Universities of Applied Sciences provide a kind of family atmosphere, that a full-time study mode is offered and that these study fields have extensive practical training within their curricula. Since all variables are self-reported, the authors cannot rule out a social desirability bias. Furthermore, an investigation into long-term behavior changes is missing. Despite these limitations, the present study contributes to improve the terms of quality development practices of health promotion programs for the German-speaking countries.

### Implications for Future Research

Although the onboarding intervention would be well-applicable to other study areas, the transfer of results and recommendations needs to be considered with caution, as findings might not be generalizable or appropriate for other fields. Therefore, students of different study areas, such as Management, Engineering and IT or Civil Engineering and Architecture should be included in health promoting onboarding intervention programs. Further research should focus on long-term studies with multiple repeated measurements, including outcomes like study success, study retention or study dropout rate.

## Conclusion

The initial study phase is a central starting point for health promoting interventions in the university setting. In order to address the students' needs, use their resources and increase acceptance, students should participate in the development of health promoting interventions. Fellow students are an important resource, thus it is vital to strengthen the relations in health promotion initiatives from the beginning of the studies. Furthermore, SoC and a salutogenic approach should be considered in intervention planning to promote the students' health, academic achievement and success. Onboarding intervention programs can benefit from alternative didactical approaches. Finally, the evaluation contributes to the development of quality within health promoting interventions and demonstrates the value of health promotion initiatives for students and, consequently, the university. Therefore, arguments for the funding and sustainable implementation of health promotion within the university setting are provided.

## Data Availability Statement

The datasets presented in this article are not readily available because the datasets generated for this study will not be made publicly available based on the paragraph in the Informed Consent that exclusively project staff have access to the collected data as well as to the source documents. Requests to access the datasets should be directed to Andrea Limarutti, a.limarutti@fh-kaernten.at.

## Ethics Statement

The studies involving human participants were reviewed and approved by Medical Ethics Committee of Carinthia, Austria (EK Nr. A35/19). The patients/participants provided their written informed consent to participate in this study.

## Author Contributions

In 2013, EM and DG conceptualized the onboarding intervention program Healthy Study Start and have been applying it in various study programs since 2013. AL conceptualized the evaluation study, was responsible for the data acquisition, interpretation of results, and drafted the paper. DG was responsible for the description of the intervention program Healthy Study Start and gave feedback on the manuscript. AL and MM were responsible for the data analysis and contributed to the drafting of the manuscript. EM participated in the conceptualization of the study and provided feedback. All authors have approved the final version of the paper.

## Conflict of Interest

The authors declare that the research was conducted in the absence of any commercial or financial relationships that could be construed as a potential conflict of interest.
